# Supplementary material for: Community pharmacies: Key players in point-of-care diagnostics for STI screening in Africa
Source: PLoS One. 2024 Dec 30;19(12):e0315191. doi: 10.1371/journal.pone.0315191 (PMC11684620; doi:10.1371/journal.pone.0315191)
Supplement: S1 Table — (PDF) [file pone.0315191.s001.pdf]

Factor	STI Infection, Frequency (%)		
	No STI<br>N=315 (70.0)<br>*n (col %)*	Have STI<br>N=135 (30.0)<br>*n (col %)*	P-Value
Age (Median (IQR)<br>≤24 years<br>25-34 years<br>≥35 years	28 (24-35)<br>87 (27.6)<br>144 (45.7)<br>84 (26.7)	28 (23-36)<br>39 (28.9)<br>59 (43.7)<br>37 (27.4)	0.682[c]<br>0.924
Gender: Male<br>Female	103 (32.7)<br>212 (67.3)	67 (49.6)<br>68 (50.4)	**0.001**
**Marital status:** Married<br>Single with no regular partner<br>Single with regular partner<br>Separated/widow	160 (50.8)<br>24 (7.6)<br>125 (39.7)<br>6 (1.9)	62 (45.9)<br>13 (9.6)<br>50 (37.0)<br>10 (7.4)	**0.028**
**Education:** Above primary<br>Primary & below	246 (78.1)<br>69 (21.9)	84 (62.2)<br>51 (37.8)	**<0.001***
**Employment:** Formal<br>Self<br>None	138 (43.8)<br>121 (38.4)<br>56 (17.8)	63 (46.7)<br>56 (41.5)<br>16 (11.9)	0.290
**Number of sex partners:** None<br>1 partner<br>2+	5 (1.6)<br>248 (78.7)<br>62 (19.7)	2 (1.5)<br>88 (65.2)<br>45 (33.3)	**0.007***
**Condom use:** Always<br>Sometimes<br>Never	17 (5.4)<br>88 (27.9)<br>210 (66.7)	3 (2.2)<br>47 (34.8)<br>85 (63.0)	0.168*
**Sex orientation:** Heterosexual<br>Homosexual<br>Bisexual<br>Unknown	268 (85.1)<br>45 (14.3)<br>0 (0.0)<br>2 (0.6)	123 (91.1)<br>10 (7.4)<br>1 (0.7)<br>1 (0.7)	0.055**
**Engagement in transactional sex**	169 (53.7)	70 (51.9)	0.672
**STI Knowledge:** High<br>Low or none	160 (50.8)<br>155 (49.2)	69 (51.1)<br>66 (48.9)	0.951
**STI symptoms present[1]**	150 (47.6)	97 (71.9)	**<0.001**
**Willingness to take prophylactic STI treatment**	303 (96.2)	125 (92.6)	0.105
**Willing to inform your partner if necessary**	287 (91.1)	113 (83.7)	**0.022**
**Used illicit drugs in the past 6 months**	10 (3.2)	6 (4.4)	0.505
**Used alcohol in the last 12 months**	71 (22.5)	48 (35.5)	**0.004**
**Used any antibiotics in the past month**	71 (22.5)	36 (26.7)	0.342*

Column percentages are presented. P-values were obtained using Pearson chi-square test except [c]P-value by rank-sum test used to compare median age, P-value by fishers exact*

- STI symptom present[1] considered participant with any symptoms including (Urethral pus discharge, Abnormal vaginal discharge, Genital swelling or Genital growth, Lower abdominal pain, Genital itching)

-Have STI considered participants with any sexually transmitted infection, i.e., HIV, Syphilis (using Abbott Bio-line Duo), Gonorrhea, Chlamydia (using Cepheid GeneXpert), Trichomoniasis (using OSOM lateral flow assay) from confirmatory laboratory tests.
